# A Prognostic Model for Acute Myeloid Leukemia Based on IL-2/STAT5 Pathway-Related Genes

**DOI:** 10.3389/fonc.2022.785899

**Published:** 2022-02-02

**Authors:** Yigang Tang, Shujun Xiao, Zhengyuan Wang, Ying Liang, Yangfei Xing, Jiale Wu, Min Lu

**Affiliations:** Shanghai Institute of Hematology, State Key Laboratory of Medical Genomics, National Research Center for Translational Medicine at Shanghai, Ruijin Hospital, Shanghai Jiao Tong University School of Medicine, Shanghai, China

**Keywords:** acute myeloid leukemia, IL-2/STAT5 pathway, prognostic model, immune infiltration, drug screening, personalized therapy

## Abstract

Accurate prognostic stratification of patients can provide guidance for personalized therapy. Many prognostic models for acute myeloid leukemia (AML) have been reported, but most have considerable inaccuracies due to contained variables with insufficient capacity of predicting survival and lack of adequate verification. Here, 235 genes strongly related to survival in AML were systematically identified through univariate Cox regression analysis of eight independent AML datasets. Pathway enrichment analysis of these 235 genes revealed that the IL-2/STAT5 signaling pathway was the most highly enriched. Through Cox proportional-hazards regression model and stepwise algorithm, we constructed a six-gene STAT5-associated signature based on the most robustly survival-related genes related to the IL-2/STAT5 signaling pathway. Good prognostic performance was observed in the training cohort (GSE37642-GPL96), and the signature was validated in seven other validation cohorts. As an independent prognostic factor, the STAT5-associated signature was positively correlated with patient age and ELN2017 risk levels. An integrated score based on these three prognostic factors had higher prognostic accuracy than the ELN2017 risk category. Characterization of immune cell infiltration indicated that impaired B-cell adaptive immunity, immunosuppressive effects, serious infection, and weakened anti-inflammatory function tended to accompany high-risk patients. Analysis of in-house clinical samples revealed that the STAT5-assocaited signature risk scores of AML patients were significantly higher than those of healthy people. Five chemotherapeutic drugs that were effective in these high-risk patients were screened *in silico*. Among the five drugs, MS.275, a known HDAC inhibitor, selectively suppressed the proliferation of cancer cells with high STAT5 phosphorylation levels *in vitro*. Taken together, the data indicate that the STAT5-associated signature is a reliable prognostic model that can be used to optimize prognostic stratification and guide personalized AML treatments.

## Introduction

Acute myeloid leukemia (AML) is the most common form of acute leukemia in adults, characterized by abnormal growth and differentiation of hematopoietic stem cells (HSCs) (1). The clinical outcomes of AML patients remain unsatisfactory, with 5-year overall survival rates of less than 50%, dropping to less than 20% for patients older than 60 years (2). The poor prognosis of AML may be attributed to the heterogeneity of therapeutic responses among patients (3) and conventional clinical therapies that have changed little over the past three decades (4). As a consequence, there is an urgent need to better stratify patients facilitating the development of personalized treatments for different patients with AML.

Signal transducer and activator of transcription 5 (STAT5), with its two isoforms STAT5A and STAT5B (5), is a key component of the janus tyrosine kinase (JAK)-signal transducer and activator of transcription (STAT) pathway (6). As a transcription factor, STAT5 can be phosphorylated upon interleukin-2 (IL-2) binding to its cognate receptor, followed by the activation of its downstream targets (7). Abnormal activation of STAT5 *via* phosphorylation frequently occurs in blast cells of patients with AML (8), where it is important for the proliferation of leukemic cells (9). High STAT5 levels are relevant to drug resistance and can desensitize BCR-ABL1^+^ leukemia cells to tyrosine kinase inhibitors (10). Additionally, phosphorylated STAT5 can suppress antitumor immunity (11) and is also engaged in the pathogenesis of chronic osteomyelitis *via* immune dysregulation (12).

Transcriptomic variables have higher predictive accuracy than clinical or genetic variables in myelodysplastic syndrome (13), and similar trends were recently observed in AML (14). However, the current widely used risk-stratification system (European Leukemia Net (ELN)2017 risk category) recommended by the National Comprehensive Cancer Network (NCCN) AML guideline (15) was constructed based on genetic variables, without considering transcriptomic changes (14). Recently, increasing numbers of prognostic models for AML based on transcriptomic data were reported, encompassing distinct biological processes such as immunity (16–18), autophagy (19), etc. However, there was no comprehensive analysis of strongly survival-related genes in AML prior to this study, which hampered the development of more accurate prognostic models based on transcriptomic data.

## Materials and Methods

### Retrieval of AML Datasets

We systematically retrieved AML datasets in the Gene Expression Omnibus (GEO) database (**Supplementary Table 1**). All datasets with more than 100 samples and available survival information were collected. The dataset with the most complete data and the largest sample size was selected when datasets overlapped. Eventually, five GEO datasets including GSE106291, GSE12417-GPL96, GSE37642-GPL96, GSE37642-GPL570, and GSE71014 were screened out for the present study (20–23). In addition, an AML cohort from The Cancer Genome Atlas (TCGA) (24), an AML cohort from Therapeutically Applicable Research to Generate Effective Treatments (TARGET), and an AML cohort from a clinical study at Oregon Health & Science University (OHSU) (3) also met the inclusion criteria and were included in this study.

Total RNA samples isolated from bone marrow mononuclear cells were used for probing gene expression levels in cohorts GSE37642-GPL96, GSE37642-GPL570, GSE71014, and TCGA. Total RNA-isolated samples from bone marrow (BM) mononuclear cells and peripheral blood (PB) mononuclear cells were used for detecting gene expression levels in cohorts GSE106291 (details unavailable), GSE12417-GPL96 (161 BM and 2 PB), OHSU (251 BM and 160 PB), and TARGET (details unavailable).

Detection of gene expression levels in different cohorts was performed on different platforms: ~20,000 encoding genes detected using Illumina HiSeq 1500 in GSE106291; ~12,000 encoding genes detected using Affymetrix Human Genome U133A Array in GSE12417-GPL96; ~12,000 encoding genes detected using Affymetrix Human Genome U133A Array in GSE37642-GPL96; ~18,000 encoding genes detected using Affymetrix Human Genome U133 Plus 2.0 Array in GSE37642-GPL570; ~20,000 encoding genes detected using HumanHT-12 V4.0 expression beadchip in GSE71014; ~20,000 encoding genes detected using Illumina HiSeq 2500 in OHSU; ~20,000 encoding genes detected using Illumina HiSeq 2000 in TARGET; and ~20,000 encoding genes detected using Illumina HiSeq 2000 in TCGA.

Processed gene expression data with respective normalization method were downloaded for bioinformatical analysis in this study. All gene expression variables were scaled to a mean value of 0 and variance equal to 1 (Z-score) in GSE10621. Normalization was performed using the variance stabilizing normalization (VSN) algorithm, and probe set expression values were calculated by the median polish method in GSE12417-GPL96. Normalization was performed using the Robust Multichip Average (RMA) method in GSE37642-GPL96 and GSE37642-GPL570. Expression values were processed with log_2_ transformation and quantile normalization in GSE71014. Normalization was performed using the conditional quantile normalization procedure in OHSU. Fragments per kilobase of exon model per million mapped fragments (FPKM) values of genes were log_2_(FPKM+1) transformed in TARGET. RNA-Seq by Expectation-Maximization (RSEM) normalized counts (norm_count) of genes were log_2_(norm_count +1) transformed in TCGA.

Normalized transcriptome data and clinical information were acquired from three different databases: GEO datasets from GEO database (http://www.ncbi.nlm.nih.gov/geo/), TCGA and TARGET datasets from the UCSC Xena database (http://xena.ucsc.edu/), as well as the OHSU dataset from cBioPortal (https://www.cbioportal.org/) (25, 26). Clinical variables of the eight cohorts were summarized in each dataset (**Supplementary Table 2**). Clinical data of GSE71014 only contained survival information. Samples without survival information or transcriptome data were excluded in each dataset. After exclusion, sample size in each cohort was as follows: GSE106291 (*n* = 250), GSE12417-GPL96 (*n* = 163), GSE37642-GPL96 (*n* = 417), GSE37642-GPL570 (*n* = 136), GSE71014 (*n* = 104), OHSU (*n* = 411), TARGET (*n* = 156), and TCGA (*n* = 151).

### Screening of Robustly Survival-Related Genes

**Figure 1:**
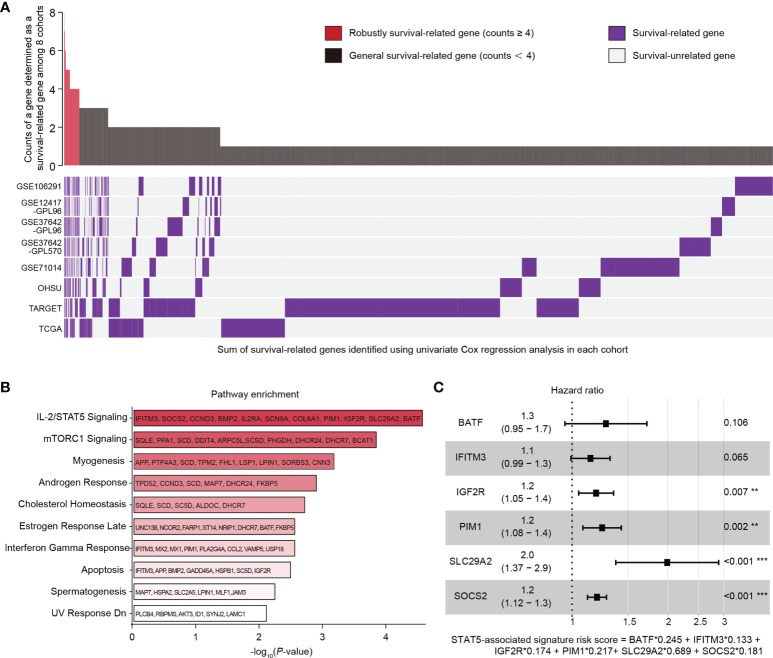
Identification of genes related to survival in AML patients and construction of a STAT5-associated signature. **(A)** Landscape of survival-related genes (purple bars) determined by univariate Cox regression analysis in eight independent datasets (lower panel). The frequency of a gene determined as a survival-related gene (purple bar) among the eight datasets was quantified (upper panel). Red bars with counts ≥4 represent the robustly survival-related genes in the upper panel. **(B)** Pathway enrichment of 235 robustly survival-related genes identified in **(A)** using the MSigDB database. The top 10 enriched pathways are shown. Annotated genes in each pathway are indicated. **(C)** Forest plot of BATF, IFITM3, IGF2R, PIM1, SLC29A2, and SOCS2. The STAT5-associated signature risk score formula was at the bottom. Error bars represent hazard ratio (HR) with 95% confidence intervals (CI).

The univariate Cox regression analysis was performed individually in eight independent AML datasets (GSE106291, GSE12417-GPL96, GSE37642-GPL96, GSE37642-GPL570, GSE71014, OHSU, TARGET, TCGA). Survival-related genes (HR > 1, *p* < 0.05) in each dataset were screened out (**Figure 1A**, purple bars). A gene which was identified as a survival-related gene in at least four datasets was defined as a robustly survival-related gene in this study. Eventually, a total of 235 robustly survival-related genes were identified (red bars in upper panel, **Figure 1A**; **Supplementary Table 3**).

### Pathway Enrichment Analysis

A total of 235 identified robustly survival-related genes (**Supplementary Table 3**) were subjected to pathway enrichment analysis using the Molecular Signatures Database (MSigDB) on Enrichr (https://maayanlab.cloud/Enrichr/). Briefly, we entered the 235 gene symbols on each row in the text-box on the Enrichr (https://maayanlab.cloud/Enrichr/) and submitted these gene symbols. We then clicked “Pathways” module in the navigation (at the top). Detailed results including enriched pathways and *p*-values could be found after clicking the icon “MSigDB Hallmark 2020”.

### Expression Distribution of STAT5A and STAT5B Among 16 Different Organs

Online website The Human Protein Atlas (https://www.proteinatlas.org/) contains information on genome-wide RNA expression profiles of human protein-coding genes in 69 human cell lines. These 69 cell lines are derived from 16 different organs including: brain, liver and gallbladder, gastrointestinal tract, pancreas, male reproductive system, kidney and urinary bladder, skin, eye, proximal digestive tract, lung, female reproductive system, endothelial, muscle, mesenchymal, lymphoid, and myeloid. Downloaded gene expression levels of STAT5A (https://www.proteinatlas.org/ENSG00000126561-STAT5A/cell+line) and STAT5B (https://www.proteinatlas.org/ENSG00000173757-STAT5B/cell+line) in the 69 cell lines were used for investigating the expression distribution of STAT5A and STAT5B among the 16 different organs.

### Protein-Protein Interaction Network

The search tool for the retrieval of interacting genes (STRING) database (http://string.embl.de/) (27) was used to visualize the associations among robustly survival-related genes related to the IL-2/STAT5 pathway. Briefly, we selected function module “Multiple proteins” in the left navigation and enter protein names including STAT5A, STAT5B, and 11 robustly survival-related genes (IFITM3, SOCS2, CCND3, BMP2, IL2RA, SCN9A, COL6A1, PIM1, IGF2R, SLC29A2, and BATF) in the search box. We selected “*Homo sapiens*” in the pull-down list of “Organism” and clicked the icon “SEARCH” under the search box. We clicked the icon “CONTINUE” in the pop-up interface and waited for a moment. The results could be found in the next pop-up interface. We downloaded the scalable vector graphic from the “Exports” module.

### Construction and Validation of a STAT5-Associated Signature

Eleven robustly survival-related genes related to the IL-2/STAT5 pathway (IFITM3, SOCS2, CCND3, BMP2, IL2RA, SCN9A, COL6A1, PIM1, IGF2R, SLC29A2, and BATF; **Figure 1B**) were used for constructing a STAT5-associated signature. In the training cohort (GSE37642-GPL96), the Cox proportional-hazards model (28) was employed to estimate the optimal weighting coefficients of these 11 robustly survival-related genes with the function *coxph* in the package “survival” (29) on the basis of maximizing the partial likelihood techniques (30, 31). For building the best performing regression model, 6 genes (BATF, IFITM3, IGF2R, PIM1, SLC29A2, SOCS2; **Figure 1C**) out of the 11 robustly survival-related genes were selected for constructing the final Cox regression model with the function *step* in R language based on the stepwise algorithm (32). The STAT5-associated signature risk score was calculated according to the sum of the coefficients multiplied by the gene expression level of each selected gene. Patients were separated into low- and high-risk groups according to the median STAT5-associated signature risk score in each cohort. The prognostic performance of this model was assessed using Kaplan-Meier analysis. The specificity of this model was evaluated using curves with area under the receiver operating characteristic (ROC) curve (AUC) values. The prognostic independence of this model was confirmed by univariate and multivariate Cox analysis.

### Improvement of the European Leukemia Net 2017 Risk Stratification System

An integrated risk model was constructed based on STAT5-associated signature, age of patients, and ELN2017 risk category using the Cox proportional-hazards model (28). This risk model was visualized by the nomogram produced by the R package “rms” (33). The predictive accuracy of this integrated risk model was assessed using calibration curves produced by the R package “rms” (33).

### Estimation of Immune Infiltration

Transcriptome data were used to estimate the composition of tumor-infiltrating immune cells based on the deconvolution algorithm of the Cell type Identification by Estimating Relative Subsets of RNA Transcripts (CIBERSORT) (34). The relative fractions of the 22 immune cell types in each sample were then determined using the function *CIBERSORT* in R language (34). An empirical *p*-value for the deconvolution was produced for each sample through Monte Carlo sampling (34). Only outputs with *p* < 0.05 were used for further analysis.

The correlations between STAT5-associated signature risk scores and fractions of tumor-infiltrating immune cells were further investigated using Spearman correlation analysis. Proportions of immune cells and stromal cells were estimated based on the immune score and stromal score, respectively. These two tumor microenvironment scores were calculated using the R package “estimate” (35).

### In-House Human Samples

Total RNA of peripheral blood mononuclear cells (from 6 healthy donors and 28 AML patients) and peripheral blood mononuclear cells (from 6 healthy donors and 6 AML patients) were collected at the Department of Hematology, the First Affiliated Hospital of Jinan University. These RNA samples were collected from June, 2020 to November, 2020 and were stored at −80°C. Peripheral blood mononuclear cells were collected from June, 2020 to November, 2020 and were stored at liquid nitrogen. Written informed consent was obtained from all patients. The Ethics Committee of the First Affiliated Hospital of Jinan University approved the study (No. KY-2020-022 in 2020).

### Real-Time Quantitative PCR Assay

The real-time quantitative PCR (RT-qPCR) assay was performed exactly as reported previously (36). Briefly, total RNA was isolated using a Total RNA Purification Kit (B518651, Sangon Biotech, Shanghai, China) following the manufacturer’s protocol. Total RNA at 1 μg was then reverse transcribed using the HiScript^®^ II QRT SuperMix for qPCR (+ gDNA wiper) (R223-01, Vazyme Biotech, Nanjing, China) following the manufacturer’s protocol. PCR was performed in triplicate using ChamQ™ SYBR^®^ qPCR Master Mix (Low ROX Premixed) (Q331-02/03, Vazyme Biotech) and a ViiA™ 7 Real-Time PCR System (Applied Biosystems, Waltham, MA, USA) under the following conditions: 10 min at 95°C, followed by 40 cycles of 95°C for 15 s and 60°C for 45 s. β-Actin was used as the housekeeping control. Primers used in this assay are as follows: BATF-forward: TATTGCCGCCCAGAAGAGC, BATF-reverse: GCTTGATCTCCTTGCGTAGAG; IFITM3-forward: AGGGACAGGAAGATGGTTGG, IFITM3-reverse: TGGGATGACGATGAGCAGAA; IGF2R-forward: CTGCCGCTATGAAATTGAGTGG, IGF2R-reverse: CGCCGCTCAGAGAACAAGTT; PIM1-forward: GAGAAGGACCGGATTTCCGAC, PIM1-reverse: CAGTCCAGGAGCCTAATGACG; SLC29A2-forward: TCAGTGCAGTCCTACAGGG, SLC29A2-reverse: GGCGTGATAAAGTACCCCAGG; SOCS2-forward: CAGATGTGCAAGGATAAGCGG, SOCS2-reverse: GCGGTTTGGTCAGATAAAGGTG; β-actin-forward: ACTTAGTTGCGTTACACCCTTTCT; β-actin-reverse: GACTGCTGTCACCTTCACCGT. Relative gene expression was normalized to β-actin and calculated by the formula: relative target gene expression = 2^−ΔCT^ (ΔCT = CT_target gene_ − CT_β-actin_). RT-qPCR cycles were uploaded as a supplementary material (**Supplementary Table 4**).

### *In Silico* Screening of Chemotherapy Drugs for the Treatment of High-Risk Patients

Clinical drug responses could be predicted using baseline gene expression levels (37). In brief, a ridge regression model was fitted for baseline gene expression levels in the 700 cell lines against the *in vitro* 138 drug half-maximal inhibitory concentration (IC_50_) estimates, and this model was then applied to the baseline tumor expression data from the clinical trial, to yield drug sensitivity estimates (37). In the present study, the ridge regression model was used to estimate the IC_50_ of 138 chemotherapeutic agents for each AML patient based on transcriptomic data, followed by 10-fold cross-validation implemented using the R package “pRRophetic” (38). A chemotherapy drug with significantly lower IC_50_ in the high-risk group was determined as a targeted drug for high-risk patients in each cohort. The frequency with which a drug was identified as a targeted drug for high-risk patients in eight cohorts was quantified. Five drugs with the highest frequencies, including bexarotene, bortezomib, erlotinib, rapamycin, and MS.275 were screened as *in silico* hits.

### Immunoblotting Assay

A specific antibody against pSTAT5 (1:1,000 dilution; #9395, Cell Signaling Technology, Danvers, MA, USA) was used to determine the levels of phosphorylated STAT5 in cancer cell lines and peripheral blood mononuclear cells. The immunoblotting assay was performed exactly as reported previously (36). The protein bands were quantified densitometrically using ImageJ software. The full uncropped immunoblotting images were uploaded as a supplementary material (**Supplementary Figure 7**).

### Cell Viability Assay

Cell viability was assessed using the Cell Counting Kit-8 (CCK8; C0005, Targetmol) following the manufacturer’s instructions. Briefly, cells were seeded in triplicate into 96-well plates at a density of 1,500–3,000 cells/well in 100 μl of medium. After treatment with the indicated chemotherapy drugs for 3 days, dye solution was added and the plates were incubated at 37°C for 3–4 h before the absorbance at 450 nm (A_450_) was measured. Cell viability was calculated by the formula: cell viability (%) = [(As-Ab)/(Ac-Ab)] × 100 where As is the absorbance of the experimental well (absorbance of cells, medium, CCK8, and wells of the test compound), Ab is the blank well absorbance (absorbance of wells containing medium and CCK8), and Ac is the control well absorbance (absorbance of wells containing cells, medium and CCK8).

### Statistical Analysis

Statistical analyses were performed using R software (version 4.0.5; R foundation for statistical computing, Vienna, Austria) and SPSS version 23.0 (IBM Corp., Armonk, NY, USA). Uni- and multivariate Cox regression analyses were conducted using the “survival” R package (29). Selected 6 genes constituting the STAT5-associated signature were separated into low- and high-expression groups based on the optimal cutoff determined using the “survminer” package (39) with the minprop variable (the minimal proportion of the observations/group) set to 20% (40). Kaplan-Meier analysis was carried out using the packages “survminer” (39) and “survival” (29), and the significance of survival differences was determined using the log-rank test. Time-dependent and time-independent receiver operating characteristic (ROC) curves were generated using the packages “timeROC” (41) and “survivalROC” (42), respectively. Nomograms and calibration curves were generated using the “rms” package (33). The statistical significance of differences between mean values of two groups was assessed using unpaired two-tailed Student’s *t*-test. Chi-squared analysis was used to evaluate the relationship between risk categories and clinicopathological parameters. The *r*- and *p*-values were determined by Spearman correlation analyses. The “pRRophetic” R package (38) was used to predict the responses to chemotherapy. The IC_50_ values of different chemotherapeutics in six cancer cell lines were estimated using the online tool IC_50_ calculator (https://www.aatbio.com/tools/ic50-calculator/). Differences with *p* < 0.05 were considered statistically significant.

## Results

### Dataset Selection and Clinical Variables of Selective Eight Datasets

Eight publicly available datasets with more than 100 samples and available survival information were selected (**Supplementary Table 1**). GSE37642-GPL96 (*n* = 417) with the largest sample size was used as a training cohort, and seven datasets including GSE106291 (*n* = 250), GSE12417-GPL96 (*n* = 163), GSE37642-GPL570 (*n* = 136), GSE71014 (*n* = 104), OHSU (*n* = 411), TARGET (*n* = 156), and TCGA (*n* = 151) were set aside as validation cohorts. Clinical variables of the eight cohorts were summarized in each dataset (**Supplementary Table 2**). Some clinically relevant features of the eight cohorts were observed. All of the patients in OHSU, for example, were under age 60 while about half of the patients in the seven other cohorts were under age 60. The ratio of men to women was close to 1:1 in the eight cohorts. Each cohort consisted mainly of M1, M2, M4, and M5 patients.

### Identification of Robustly Survival-Related Genes in AML

Univariate Cox regression analysis was performed individually in eight independent AML cohorts (GSE106291, GSE12417-GPL96, GSE37642-GPL96, GSE37642-GPL570, GSE71014, OHSU, TARGET, and TCGA; **Figure 1A**) and survival-related genes (HR > 1, *p* < 0.05) in each cohort were identified (**Figure 1A**; lower panel, purple bars). A gene which was determined to be survival related in at least four cohorts was defined as a robustly survival-related gene in the present study (**Figure 1A**; upper panel, red bars). A total of 235 identified robustly survival-related genes (**Figure 1A**; upper panel, red bars; **Supplementary Table 3**) were then subjected to pathway enrichment analysis using the Molecular Signatures Database (MSigDB), and the IL-2/STAT5 pathway was the most highly enriched item with 11 annotated genes including IFITM3, SOCS2, CCND3, BMP2, IL2RA, SCN9A, COL6A1, PIM1, IGF2R, SLC29A2, and BATF (**Figure 1B**). In addition, the gene expression levels of STAT5A and STAT5B were found to be organ specific and were significantly higher in cancer cells derived from lymphoid and myeloid organs (**Supplementary Figure 1A**). However, there was no difference in the expression levels of the two STAT5 genes between cancer cells derived from lymphoid and myeloid (**Supplementary Figure 1A**). The abnormal STAT5 expression pattern suggested that genes involved in STAT5-associated pathways might be alternative prognostic biomarkers for hematological malignancies.

### Construction of a STAT5-Associated Signature

Eleven identified robustly survival-related genes annotated in the IL-2/STAT5 pathway (**Supplementary Figure S1B**, text in white) were subjected to construct a STAT5-associated signature using Cox proportional-hazards regression model and stepwise algorithm in the training cohort (GSE37642-GPL96) (Details seen in the method section; **Figure 1C**). The STAT5-associated signature was described using the formula risk score = Exp_BATF_ ∗ 0.245 + Exp_IFITM3_ ∗ 0.133 + Exp_IGF2R_ ∗ 0.174 + Exp_PIM1_ ∗ 0.217 + Exp_SLC29A2_ ∗ 0.689 + Exp_SOCS2_ ∗ 0.181 (**Figure 1C**). The STAT5-associated signature risk score of each AML patient was then calculated and used to stratify patients into low- and high-risk groups according to the median risk score in each cohort.

The prognostic performance of the selected 6 genes that constitute this model was assessed using Kaplan-Meier analysis after classification into low- and high-expression groups in the training cohort (GSE37642-GPL96) (**Supplementary Figures 1C–H**). AML patients with high expression of any one of the six genes had significantly shorter overall survival (**Supplementary Figures 1C–H**). Among the six genes, SLC29A2 with the biggest weighting coefficient in the STAT5-associated signature might be the most significant prognostic marker to stratify AML patients (**Figure 1C**).

### Performance of the STAT5-Associated Signature

The prognostic performance of the signature was next assessed in the training cohort (GSE37642-GPL96), as well as the seven validation cohorts GSE106291, GSE12417-GPL96, GSE37642-GPL570, GSE71014, OHSU, TARGET, and TCGA. The relationship between STAT5-associated signature risk scores and survival status of patients in the cohorts is shown in **Supplementary Figures 2A–H**. Kaplan-Meier analysis indicated that patients in the high-risk group had significantly shorter overall survival in the training cohort (GSE37642-GPL96, *p* = 7.783e−10, **Table 1**; **Supplementary Figure 3A**). In line with the performance in the training cohort (GSE37642-GPL96), we found that the STAT5-associated signature also worked well in external validation cohorts, where patients in the high-risk group had shorter overall survival (GSE106291, *p* = 3.654e−04, **Table 1** and **Supplementary Figure 3B**; GSE12417-GPL96, *p* = 1.282e−02, **Table 1** and **Supplementary Figure 3C**; GSE37642-GPL570, *p* = 7.086e−04, **Table 1** and **Supplementary Figure 3D**; GSE71014, *p* = 2.618e−03, **Table 1** and **Supplementary Figure 3E**; OHSU, *p* = 1.478e−05, **Table 1** and **Supplementary Figure 3F**; TARGET, *p* = 8.45e−04, **Table 1** and **Supplementary Figure 3G** and TCGA, *p* = 8.631e−05, **Table 1** and **Supplementary Figure 3H**). The time-independent AUC value of this model reached 0.705 in the training cohort (GSE37642-GPL96), with time-dependent AUC values for 1-, 3- and 5-year survival of 0.705, 0.731, and 0.703, respectively (**Table 1**; **Supplementary Figure 4A**). Moreover, the STAT5-associated signature also showed high predictive accuracy in most of the validation cohorts (GSE106291, **Table 1** and **Supplementary Figure 4B**; GSE12417-GPL96, **Table 1** and **Supplementary Figure 4C**; GSE37642-GPL570, **Table 1** and **Supplementary Figure 4D**; GSE71014, **Table 1** and **Supplementary Figure 4E**; OHSU, **Table 1** and **Supplementary Figure 4F**; TARGET, **Table 1** and **Supplementary Figure 4G**; and TCGA, **Table 1** and **Supplementary Figure 4H**).

**Table 1:** Estimation of STAT5-associated signature risk scores: Kaplan-Meier analysis and AUG of time-independent and time-dependent ROC curves in the 8 cohorts.

	Prognostic Performance (Kaplan-Meier analysis)		Predictive Accuracy (AUG of ROC curves)			
	Higher Risk Score Indicated Poorer Prognosis?	*p*	Overall	1 Year	3 Years	5 Years
GSE37642-GPL96	Yes	**0.0000**	0.705	0.705	0.731	0.703
GSE10691	Yes	**0.0004**	0.675	0.677	0.627	0.691
GSE12417-GPL96	Yes	**0.0128**	0.723	0.726	0.668	NA
GSE37642-	Yes	**0.0007**	0.674	0.675	0.633	0.677
GPL570						
GSE71014	Yes	**0.0026**	0.720	0.734	0.746	0.729
OHSU	Yes	**0.0000**	0.667	0.662	0.616	0.538
TARGET	Yes	**0.0008**	0.608	0.608	0.662	0.651
TCGA	Yes	**0.0001**	0.708	0.698	0.678	0.660

Bold values indicate p < 0.05.

### Evaluating Prognostic Independence of the STAT5-Associated Signature

**Figure 2:**
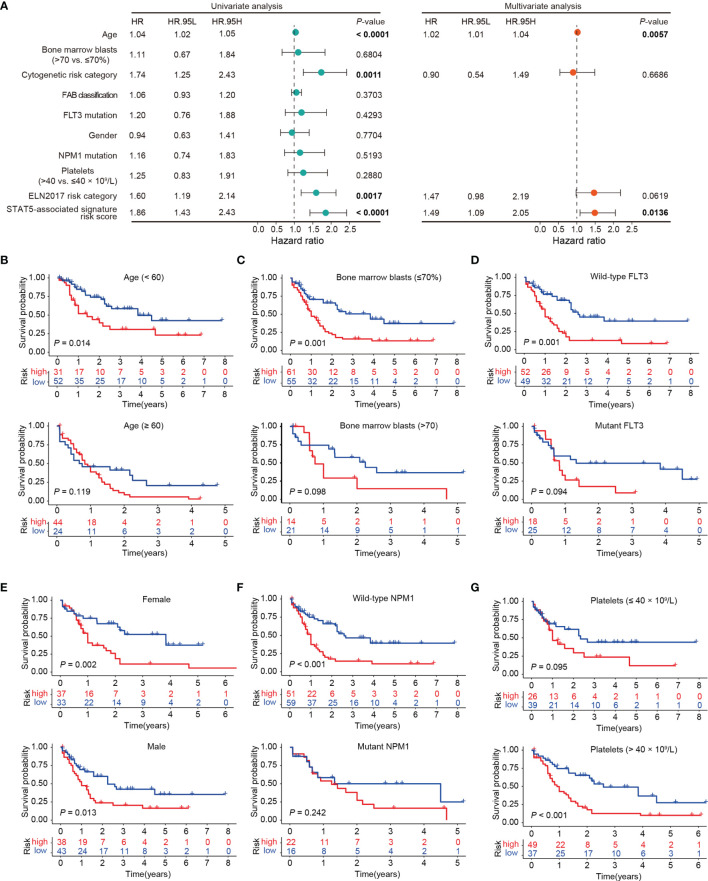
Prognostic independence of the STAT5-associated signature. **(A)** Univariate and multivariate Cox regression analyses of the STAT5-associated signature and clinicopathological variables. Error bars represent hazard ratio (HR) with 95% confidence intervals (CI). **(B–G)** Kaplan-Meier survival analysis of subgroups stratified by age <60 and ≥60 **(B)**, bone marrow blasts ≤70% and >70% **(C)**, FLT3 wild-type and mutant subgroups **(D)**, female and male subgroups **(E)**, NPM1 wild-type and mutant subgroups **(F)**, platelets ≤40 and >40 × 10^9^/L **(G)**, respectively.

The prognostic independence of the STAT5-associated signature was assessed through uni- and multivariate Cox regression analyses in the training cohort (GSE37642-GPL96) and all of the validation cohorts except for GSE71014, which lacked clinicopathological variables (**Supplementary Table S5**). In univariate Cox analysis, age, cytogenetic risk category, ELN2017 risk category, and STAT5-associated signature risk score were significantly correlated with overall survival of AML patients (**Figure 2A**). In multivariate Cox analysis, the STAT5-associated signature was proved to be an independent predictor of survival in the TCGA cohort, with HR of 1.49 (1.09–2.05, *p* = 0.0136, **Figure 2A**). The predictive independence was also confirmed in other validation cohorts and corresponding values were 1.67 (1.39–2.00; *p* < 0.001) in GSE37642-GPL96, 1.05 (1.02–1.09; *p* = 0.003) in GSE106291, 1.21 (1.13–1.31; *p* < 0.001) in GSE12417-GPL96, 2.66 (1.66–4.25; *p* < 0.001) in GSE37642-GPL570, and 1.01 (1.00–1.03; *p* = 0.035) in OHSU (**Supplementary Table S5**). In low- and high-risk groups, subgroup survival analyses by ages, percentage bone marrow blasts, FLT3 status, gender, NPM1 status, and platelet counts were performed in the TCGA cohort (**Figures 2B–G**). The STAT5-associated signature was also a promising prognostic predictor of overall survival in subgroups of patients in the TCGA cohort (**Figures 2B–G**). These results indicated that the STAT5-associated signature was an independent prognostic biomarker for AML.

### Construction of an Integrated Risk Score

In multivariate analyses, the STAT5-associated signature risk score and age of patients were independent predictors of survival, respectively (**Figure 2A** and **Supplementary Table S5**). To improve the predictive accuracy of the ELN2017 risk categories, the STAT5-associated signature risk score, age of patients, and ELN2017 risk category were integrated into an integrated score to predict the 1-, 3-, and 5-year survival probabilities in the training cohort (GSE37642-GPL96), which was visualized by a nomogram (**Supplementary Figure 5A**). The calibration curves were used to assess the predictive accuracy of the integrated score and revealed that the predicted survival 1-, 3-, and 5-year probabilities by integrated score were in good accordance with the corresponding actual survival probabilities (The higher the overlap between the red lines and black dashed lines, the more accurate the integrated score; **Supplementary Figure 5B**). The integrated scores with higher AUC values had higher predictive accuracy than the ELN2017 risk category alone (**Supplementary Figures 5C, D**). Furthermore, the advantage of the integrated score was confirmed in two additional validation cohorts (**Supplementary Figures 5E, F**).

### Distribution of STAT5-Associated Signature Risk Scores in Different Subgroups

**Figure 3:**
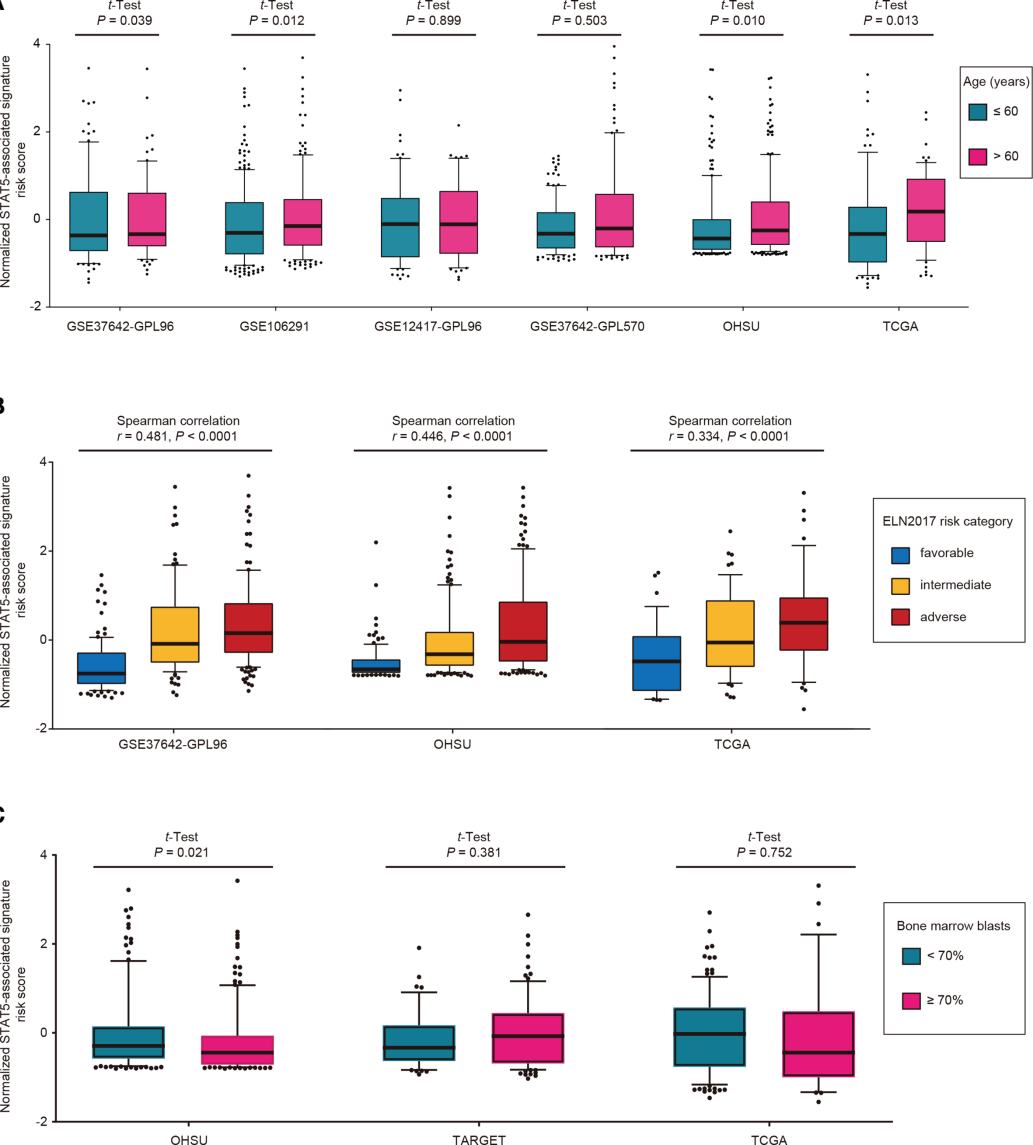
Distribution of STAT5-associated signature risk scores for different subgroups in the indicated cohorts. **(A)** Age, **(B)** ELN2017 risk category, and **(C)** bone marrow blasts. Among the total 8 cohorts, some cohorts were excluded due to inaccessibility of corresponding variables.

The distribution of STAT5-associated signature risk scores in diverse clinical and genetic risk subgroups was also investigated. Patients with an age of >60 years had significantly higher STAT5-associated signature risk scores compared with those with an age of ≤60 years in the training cohort (GSE37642-GPL96, *p* = 0.039; **Figure 3A**). Similar associations were also observed in GSE106291 (*p* = 0.012), OHSU (*p* = 0.010), and TCGA (*p* = 0.013) (**Figure 3A**). The STAT5-associated signature risk scores correlated well with the ELN2017 risk categories and increased along with the unfavorable ELN2017 risk levels in the training cohort (GSE37642-GPL96, *r* = 0.481, *p* < 0.0001, Spearman correlation, **Figure 3B**). This correlation was confirmed in two other validation cohorts (OHSU, *r* = 0.446, *p* < 0.0001, Spearman correlation; TCGA, *r* = 0.334, *p* < 0.0001, Spearman correlation; **Figure 3B**). However, no correlation between STAT5-associated signature risk scores and percentage of bone marrow blasts was observed in the TARGET and TCGA cohorts, except for the OHSU cohort (**Figure 3C**).

### Characterization of Immune Cell Infiltration in Distinct STAT5-Associated Risk Groups

**Figure 4:**
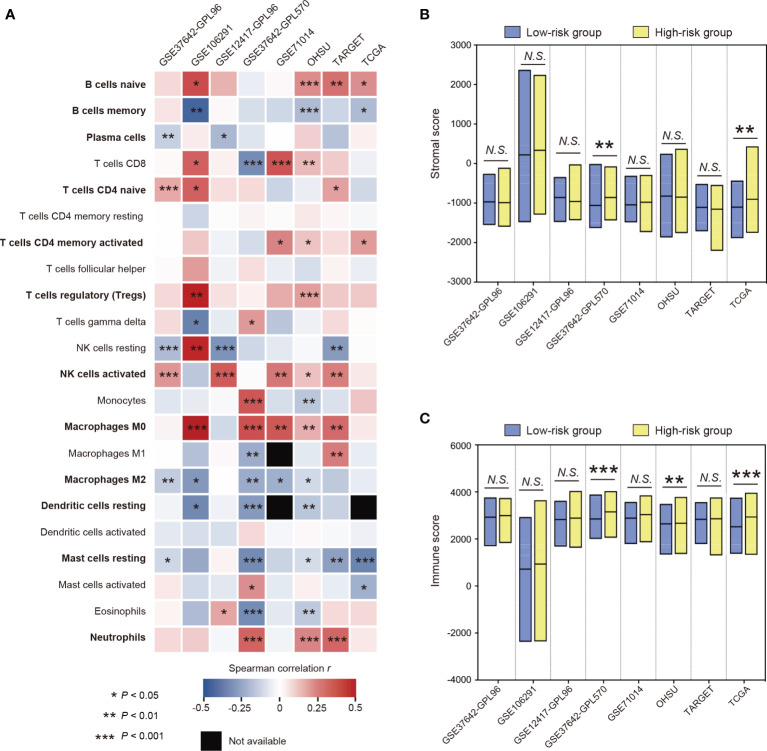
The characterization of immune cell infiltration based on the STAT5-associated signature. **(A)** Heatmap showing the relationship between fractions of tumor-infiltrating immune cells and STAT5-associated signature risk scores in each cohort. Twelve immune cell types that have strong correlations with STAT5-associated signature risk scores were highlighted in bold font. **(B, C)** Floating bars showing the differential composition of stromal cells **(B)** and immune cells **(C)** in the low- and high-risk groups. *P < 0.05, **P < 0.01, ***P < 0.001, N.S., not significant.

The characterization of immune-cell infiltration in distinct STAT5-associated risk groups was explored. The fractions of tumor-infiltrating immune cells were determined using CIBERSORT (34). The correlations between the fractions of tumor-infiltrating immune cells and STAT5-associated signature risk scores were assessed by Spearman correlation analysis in the training cohort (GSE37642-GPL96) and seven other cohorts (**Figure 4A**). The STAT5-associated signature risk scores were positively correlated with fractions of naïve B cells, naïve CD4^+^ T cells, activated CD4^+^ memory T cells, regulatory T cells (Tregs), activated NK cells, M0 macrophages, and neutrophils (**Figure 4A**). On the contrary, the STAT5-associated signature risk scores were negatively correlated with fractions of memory B cells, plasma cells, M2 marcophages, resting dendritic cells, resting mast cells (**Figure 4A**). In terms of the tumor microenvironment, patients in high-risk groups had significantly higher fractions of stromal cells and immune cells in some cohorts (**Figures 4B, C**).

### Validation of the STAT5-Associated Signature by Analysis of In-House Clinical Samples

**Figure 5:**
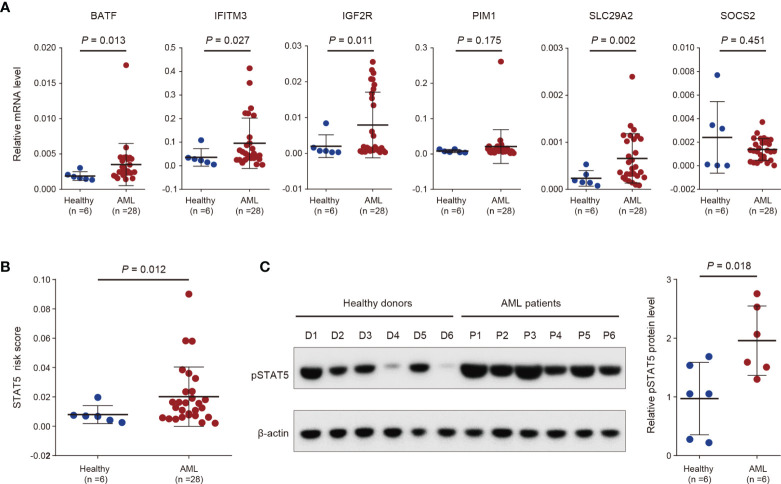
Validation of the STAT5-associated signature by analysis of in-house clinical samples. **(A)** Scatter dot plots showing gene expression levels of the indicated 6 genes used for constructing the STAT5-associated signature in peripheral blood mononuclear cells from 6 healthy donors and 28 AML patients. **(B)** Scatter dot plots showing the STAT5-associated signature risk scores of 6 healthy donors and 28 AML patients. **(C)** Protein levels of phosphorylated STAT5 (pSTAT5) in peripheral blood mononuclear cells from 6 healthy donors and 6 AML patients (left panel). Scatter dot plots showing the statistical analysis of quantified pSTAT5 levels in the left panel (right panel). Error bars in **(A–C)** represent means with standard deviation (SD). *p*-values in **(A–C)** were determined using two-tailed Student’s *t*-test.

To validate the STAT5-associated signature, we detected the gene expression of the selected 6 genes for constructing the signature in peripheral blood mononuclear cells derived from 6 healthy donors and 28 AML patients using RT-qPCR (**Figure 5A**). Gene expression levels of BATF, IFITM3, IGF2R, and SLCA29A2 were significantly higher in primary cells from AML patients than from healthy donors (**Figure 5A**). No difference in gene expression levels of PIM1 and SOCS2 was observed between primary cells from healthy donors and AML patients (**Figure 5A**). The STAT5-associated signature risk scores of all people were calculated based on the gene expression levels of the 6 genes in **Figure 5A**. AML patients had significantly higher STAT5-associated signature risk scores than healthy donors (**Figure 5B**). Phosphorylation of STAT5 is a prerequisite for activation of STAT5-associated pathways (43). As expected, phosphorylated STAT5 (pSTAT5) levels were higher in peripheral blood mononuclear cells from AML patients than healthy donors (**Figure 5C**).

### *In Silico* Screening of Chemotherapy Drugs for Treatment of High-Risk AML Patients

**Figure 6:**
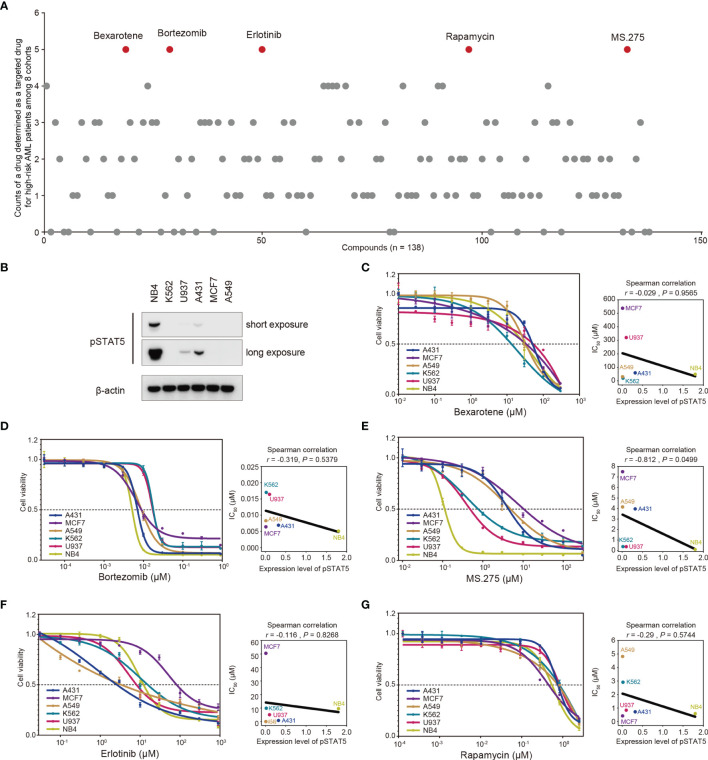
*In silico* screening of chemotherapy drugs for treatment of high-risk AML patients. **(A)** Half-maximal inhibitory concentration (IC_50_) of 138 chemotherapeutic agents for each patient was estimated based on the transcriptomic data. A drug with significantly lower IC_50_ in the high-risk group was determined as a targeted drug for high-risk patients in each cohort. The frequency with which a drug was determined as a targeted drug for high-risk patients among eight cohorts was quantified. Five drugs with the highest frequencies were screened out (red dots). **(B)** Baselines of phosphorylated STAT5 (pSTAT5) protein levels in six cancer cell lines. **(C–G)** Six cancer cell lines with different protein levels of phosphorylated STAT5 were treated with the indicated drugs for three days, followed by determination of cell viability. The correlations between IC_50_ values and protein levels of phosphorylated STAT5 were determined by Spearman correlation analysis.

Half-maximal inhibitory concentrations (IC_50_) of 138 chemotherapeutic agents were estimated for each patient based on the transcriptomic data using the “pRRophetic” R package (38) (details seen in the *Method* section). A drug with significantly lower IC_50_ in the high-risk group was determined as a targeted drug for high-risk patients in each cohort. The frequency with which a drug was determined as a targeted drug for high-risk patients among eight cohorts were quantified (**Figure 6A**), and five drugs with the highest frequencies were selected as screening hits, including bexarotene, bortezomib, erlotinib, rapamycin, and MS.275 (**Figure 6A** and **Supplementary Figure 6A**).

Further exploration of the underlying mechanism through which these five drugs targeted high-risk patients was also conducted. Phosphorylation of STAT5 is a prerequisite for activation of STAT5-associated pathways (43). Accordingly, the sensitivity of cell lines with different protein levels of phosphorylated STAT5 (**Figure 6B**) to these five drugs was determined in a cell proliferation assay (**Figures 6C–G**). The cell proliferation assay showed that the IC_50_ values of MS.275 were negatively correlated with the protein levels of phosphorylated STAT5 in six cancer cell lines (*r* = −0.812, *p* = 0.0499; **Figure 6E**). However, no correlation was observed for four other drugs (**Figures 6C, D, F, G**). Overall, MS.275 might be a promising chemotherapy drug for the treatment of high-risk patients by targeting STAT5-associated pathways.

## Discussion

In the present study, 235 robustly survival-related genes for AML were systematically identified through univariate Cox regression analysis of eight independent AML datasets. Pathway enrichment analysis with these 235 genes determined IL-2/STAT5 signaling pathway was the most highly enriched. In addition, it was reported that other enriched pathways including mechanistic target of rapamycin complex 1 (mTORC1) signaling pathway, androgen response, cholesterol homeostasis, estrogen response, and interferon gamma response were related to AML (44–48). Prognostic models based on these gene pathways might be alternative candidates for predicting prognosis of AML patients.

The STAT5-associated prognostic signature for AML was constructed based on the genes BATF, IFITM3, IGF2R, PIM1, SLC29A2, and SOCS2. BATF, basic leucine zipper transcription factor ATF-like, is an important positive transcriptional regulator of the immune system that is particularly important in classical dendritic cell development, T follicular helper cell function and antibody production (49). IFITM3, interferon-induced transmembrane protein, plays a key role in cancer cell growth and maintenance, and is a marker of poor prognosis with high expression in many cancers, including AML (50). IGF2R, insulin-like growth factor 2 receptor, is currently considered a tumor suppressor gene, but it is upregulated and correlated with poor prognosis in cervical cancer (51) and glioblastomas (52). PIM1, proviral insertion site in murine leukemia virus (PIM) kinase 1, belongs to the PIM kinase family and has been implicated in the control of cancer cell proliferation, migration, and apoptosis, particularly in prostate cancer and leukemia (53). SLC29A2, solute carrier family 29 member 2, is aberrantly upregulated and is a survival predictor in both hepatocellular carcinoma (54) and mantle cell lymphoma (55). SOCS2, suppressor of cytokine signaling-2, is highly upregulated and has tumor-promoting functions in the advanced stage of chronic myeloid leukemia (56) and in high-grade anal intraepithelial lesions (57). Furthermore, upregulation of SOCS2 is recognized as a potential prognostic marker for prostate cancer (58).

The good performance of the STAT5-associated signature was reproduced in most of the validation cohorts. Moreover, this signature was proven to be an independent prognostic factor upon multivariate Cox regression analysis and stratified survival analyses of several clinical characteristics. These results suggest that the STAT5-associated prognostic model may help predict the survival of AML patients.

It was reported that transcriptomic variables have higher predictive accuracy than genetic variables (13). However, the widely used clinical risk stratification system for AML, ELN2017, was constructed based on genetic and not transcriptomic variables (15). To complement this risk-assessment tool, an integrated score encompassing ELN2017 risk stratification, STAT5-associated signature risk scores and age of patients was constructed in the training cohort (GSE37642-GPL96). The STAT5-associated signature could improve the prognostic accuracy of ELN2017 risk categories in the training cohort (GSE37642-GPL96) as well as in two other independent cohorts.

Persistently phosphorylated STAT5 was found to suppress antitumor immunity (11). This suggests that immunological features also need to be investigated in myeloid neoplasms, since they will likely improve our knowledge of the underlying pathogenesis and inform novel therapies (18). Here, we characterized immune cell infiltration based on STAT5-associated risk stratification. The STAT5-associated signature risk scores were positively correlated with fractions of naïve B cells, and negatively correlated with fractions of memory B cells and plasma cells, which suggested impaired B-cell adaptive immunity in patients with high STAT5-associated signature risk scores (59, 60). Fractions of regulatory T cells (Tregs) and naïve CD4^+^ T cells were found to be positively correlated with STAT5-associated signature risk scores, which implied immunosuppressive effects in the patients with high STAT5-associated signature risk scores (61, 62). Along with increasing STAT5-associated signature risk scores, we observed increasing neutrophils, increasing activated CD4^+^ memory T cells, decreasing resting mast cells, and decreasing resting dendritic cells, which indicated severe infection in the patients with high STAT5-associated signature risk scores (63–66). At the same time, the anti-inflammatory function of high-risk patients might be weakened due to negative association of STAT5-associated signature risk scores with fractions of M2 macrophages and positive association of STAT5-associated signature risk scores with M0 macrophages (67). Unexpectedly, fractions of activated NK cells were positively correlated with STAT5-associated signature risk scores. However, similar results were observed in another independent study (40), which might indicate tumor escape *via* defective expression of NK cell-triggering receptors by leukemic cells (68).

Chemotherapy remains the main treatment strategy for AML (69), and screening more effective chemotherapy drugs for high-risk patients might be a quick and economical strategy for improving survival. To potentially improve the prognosis of high-risk patients, five chemotherapy drugs that were likely to be effective in high-risk patients were selected through *in silico* screening. The underlying mechanisms through which these five drugs target the high-risk patients were then investigated using cell viability assays. Among the five drugs, MS.275 selectively suppressed the cell lines with highly phosphorylated STAT5. This result suggested that MS.275 might be a promising drug for the treatment of high-risk AML patients by targeting STAT5-associated pathways. MS.275 (Entinostat) is an oral class I histone deacetylase (HDAC) inhibitor that blocks cell proliferation and promotes apoptosis in breast cancer (70). The antitumor activity of MS.275 in AML was also reported, including the induction of robust differentiation of AML cell lines (71), inducing apoptosis in AML cell lines (72), and inhibited disease maintenance in a mouse model of AML (73). Clinical trials of MS.275 for the treatment of hematological cancers including AML were also performed by different groups (NCT00015925, NCT01159301, NCT01132573, NCT00313586, NCT01305499, NCT00462605, NCT00101179, NCT01383447). These concerted efforts will enrich the therapy regimen for AML in the clinic, and hopefully improve the prognosis of high-risk patients.

However, there are also some limitations to the current study. In the subgroup analysis used to validate the prognostic independence of this model, the difference was not statistically significant due to an insufficient number of patients in some subgroups, such as the mutant NPM1 subgroup. The underlying mechanisms through which the five chemotherapy drugs other than MS.275 target AML in high-risk patients are still unknown. Potential biases of this model exist due to heterogeneity of patients, therapy regimens, and disease stage. Additionally, this is a retrospective study with a few experiments, so the findings remain to be further validated in both the laboratory and the clinic.

In conclusion, we comprehensively analyzed the genes that are most strongly related to survival in AML. Pathway enrichment analysis of these robustly survival-related genes indicated that IL-2/STAT5 is the most highly enriched signaling pathway. A STAT5-associated signature was constructed on the basis of robustly survival-related genes related to the IL-2/STAT5 signaling pathway. The signature could independently predict survival of AML patients, and our prognostic model might complement and improve the current risk system based on genetic variables, such as the ELN2017 risk categories. The immune infiltration was also investigated based on the risk phenotype, which will contribute to immunotherapy of high-risk patients in the future. Analysis of in-house clinical samples revealed that the STAT5-assocaited signature risk scores of AML patients were significantly higher than those of healthy people. MS.275, a known HDAC inhibitor, was demonstrated as a targeted drug for high-risk patients by interfering with STAT5-associated pathways. This reliable model could be used for prognostic assessment and guidance for precision therapy for AML.

## Data Availability Statement

The original contributions presented in the study are included in the article/**Supplementary Material**. Further inquiries can be directed to the corresponding author.

## Ethics Statement

The studies involving human participants were reviewed and approved by IRB of the First Affiliated Hospital of Jinan University. The patients/participants provided their written informed consent to participate in this study.

## Author Contributions

ML and YT designed this work. YT, ZW, YL, YX, JW, and SX retrieved the data and conducted the analyses. YT, SX, YL, YX, JW, and ZW performed the experiment. ML, YT, and SX wrote this manuscript. All authors listed have made a substantial, direct, and intellectual contribution to the work and approved it for publication.

## Funding

This work was funded by the National Natural Science Foundation of China (NSFC 81900157 & 82102756), the China Postdoctoral Science Foundation (2021M692111 & 2018M640399), and the SJTU Trans-med Awards Research and the Foundation of the National Facility for Translational Medicine (Shanghai) (TMSK-2020-003).

## Conflict of Interest

The authors declare that the research was conducted in the absence of any commercial or financial relationships that could be construed as a potential conflict of interest.

## Publisher’s Note

All claims expressed in this article are solely those of the authors and do not necessarily represent those of their affiliated organizations, or those of the publisher, the editors and the reviewers. Any product that may be evaluated in this article, or claim that may be made by its manufacturer, is not guaranteed or endorsed by the publisher.

## Supplementary Material

Supplementary Figure 1 Identification of genes related to survival in AML patients and construction of a STAT5-associated signature. **(A)** The gene expression levels of STAT5A and STAT5B in 69 cancer cell lines derived from 16 distinct organs. Each dot represents a cancer cell line. *P* values were determined using two-tailed Student’s *t*-test. **(B)** The protein-protein interaction (PPI) network of robustly survival-related genes (text in white) mapping to the IL-2/STAT5 pathway. **(C–H)** Kaplan–Meier survival analysis of low- and high-expression groups of BATF, IFITM3, IGF2R, PIM1, SLC29A2, and SOCS2.

Supplementary Figure 2 The relationship between STAT5-associated signature risk scores and survival status. **(A–H)** STAT5-associated signature risk scores arrangement and survival status analyses in GSE37642-GPL96 **(A)**, GSE106291 **(B)**, GSE12417-GPL96 **(C)**, GSE37642-GPL570 **(D)**, GSE71014 **(E)**, OHSU **(F)**, TARGET **(G)**, and TCGA **(H)**.

Supplementary Figure 3 Kaplan–Meier survival analyses. **(A–H)** Kaplan–Meier curves for overall survival stratified by different risk levels in GSE37642-GPL96 **(A)**, GSE106291 **(B)**, GSE12417-GPL96 **(C)**, GSE37642-GPL570 **(D)**, GSE71014 **(E)**, OHSU **(F)**, TARGET **(G)**, and TCGA **(H)**.

Supplementary Figure 4 Time-independent and time-dependent ROC analyses. **(A–H)** AUC of time-independent (left) and time-dependent (right) ROC curves of STAT5-associated signature in GSE37642-GPL96 **(A)**, GSE106291 **(B)**, GSE12417-GPL96 **(C)**, GSE37642-GPL570 **(D)**, GSE71014 **(E)**, OHSU **(F)**, TARGET **(G)**, and TCGA **(H)**.

Supplementary Figure 5 Construction of an integrated risk score. **(A)** Nomogram visualizing the integrated risk model constructed based on the STAT5-associated signature risk score, patient age, and ELN2017 risk category in the training cohort (GSE37642-GPL96). **(B)** Calibration curves of the nomogram in terms of agreement between predicted and observed 1-year, 3-year, 5-year survival in the training cohort (GSE37642-GPL96). Error bars represent actual overall survival probability with 95% confidence intervals (CI). **(C)** Comparison of the time-dependent ROC curves of the integrated risk score and its component single risk categories in the training cohort (GSE37642-GPL96). **(D–F)** Comparison of the time-independent ROC curves of the integrated risk score and its component single risk categories in GSE37642-GPL96 **(D)**, OHSU **(E)**, and TCGA **(F)**.

Supplementary Figure 6
*In silico* screening of chemotherapy drugs for treatment of high-risk AML patients. **(A)** Estimated IC_50_ for the five hits from in the indicated cohorts.
