# Supplementary figures and images for: A Prognostic Model for Acute Myeloid Leukemia Based on IL-2/STAT5 Pathway-Related Genes
Source: Front Oncol. 2022 Feb 2;12:785899. doi: 10.3389/fonc.2022.785899 (PMC8847395; doi:10.3389/fonc.2022.785899)

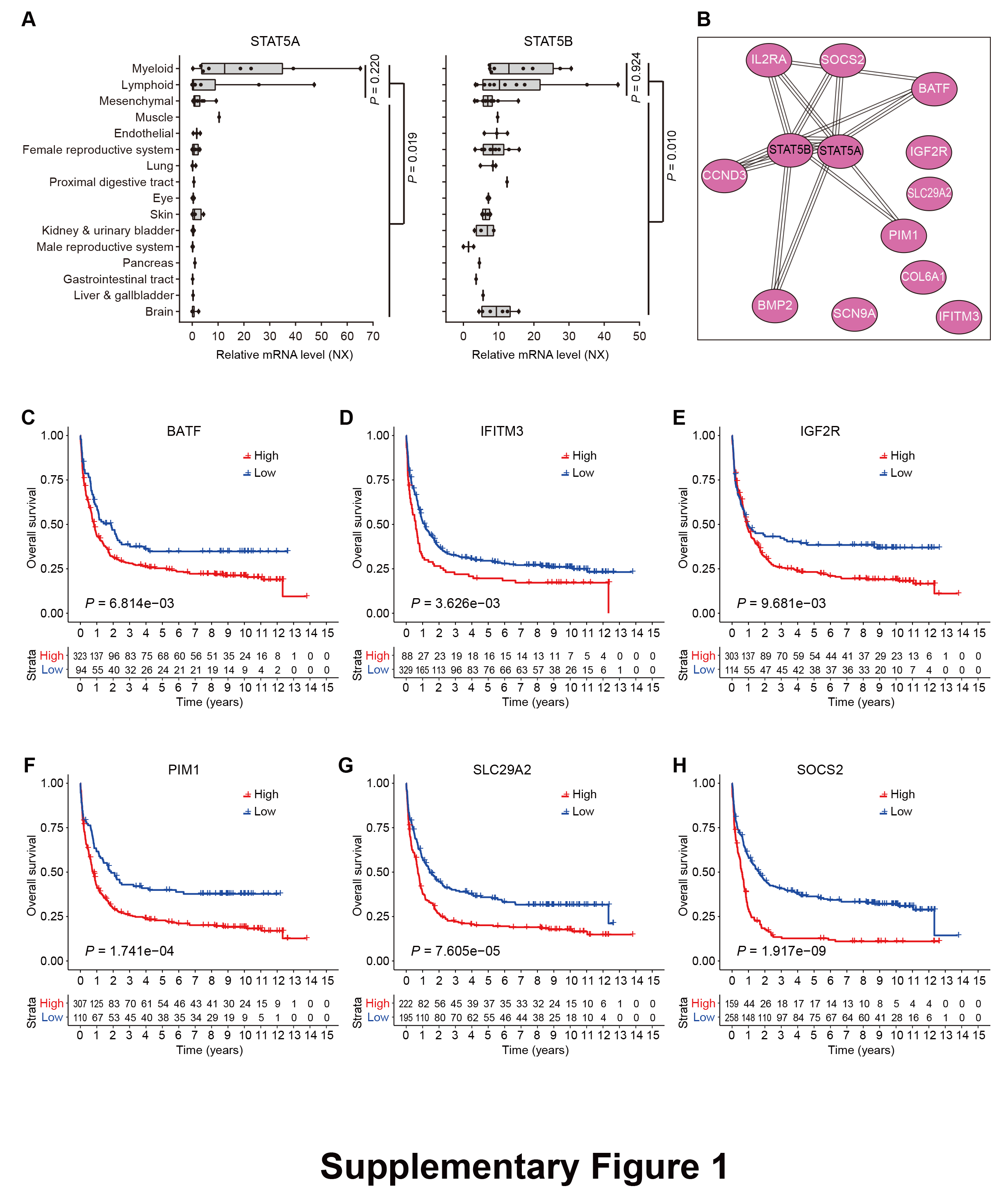

Supplement: Supplementary Figure 1 — Identification of genes related to survival in AML patients and construction of a STAT5-associated signature. (A) The gene expression levels of STAT5A and STAT5B in 69 cancer cell lines derived from 16 distinct organs. Each dot represents a cancer cell line. P values were determined using two-tailed Student’s t-test. (B) The protein-protein interaction (PPI) network of robustly survival-related genes (text in white) mapping to the IL-2/STAT5 pathway. (C–H) Kaplan–Meier survival analysis of low- and high-expression groups of BATF, IFITM3, IGF2R, PIM1, SLC29A2, and SOCS2. [file Image_1.tif]

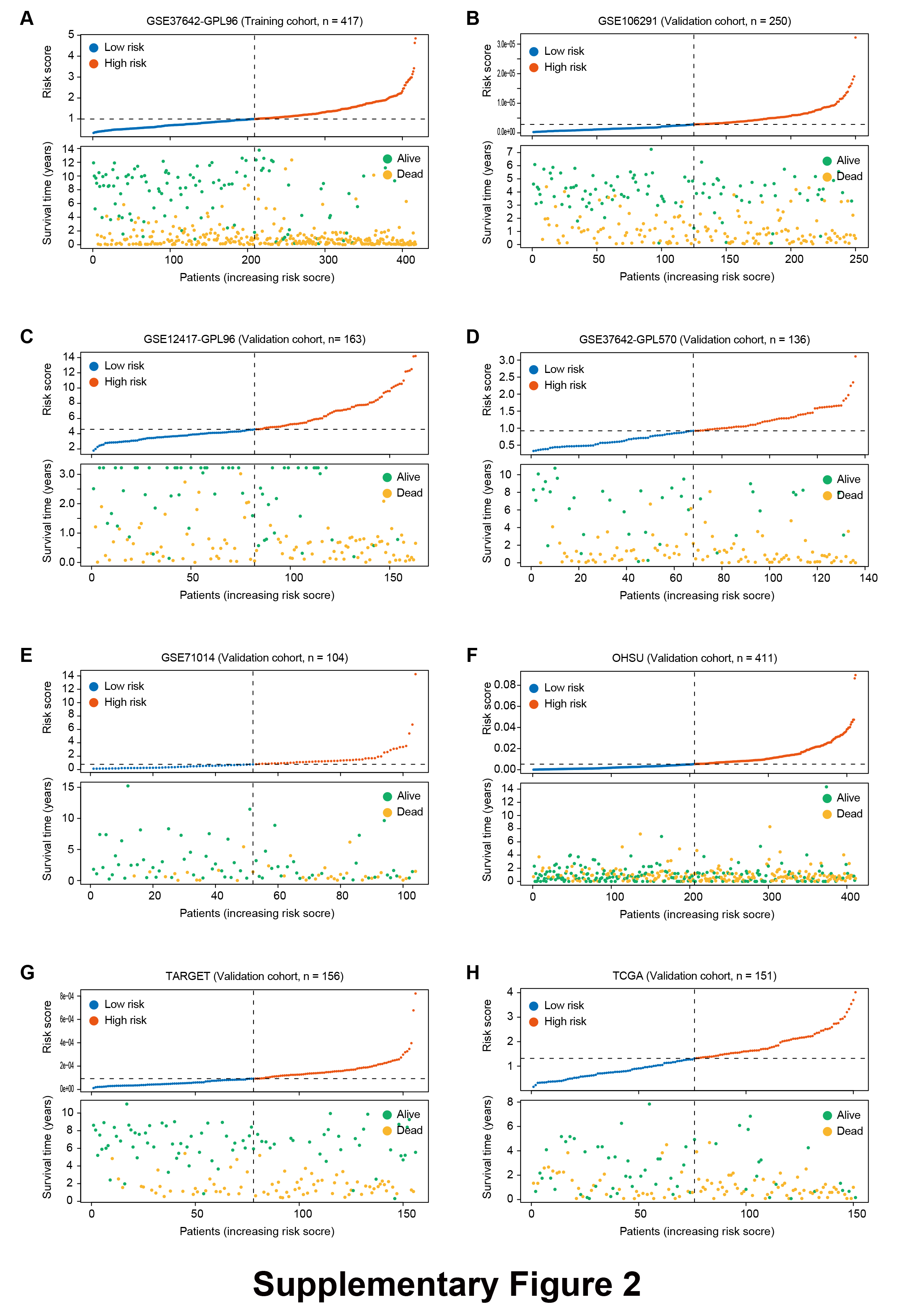

Supplement: Supplementary Figure 2 — The relationship between STAT5-associated signature risk scores and survival status. (A–H) STAT5-associated signature risk scores arrangement and survival status analyses in GSE37642-GPL96 (A), GSE106291 (B), GSE12417-GPL96 (C), GSE37642-GPL570 (D), GSE71014 (E), OHSU (F), TARGET (G), and TCGA (H). [file Image_2.tif]

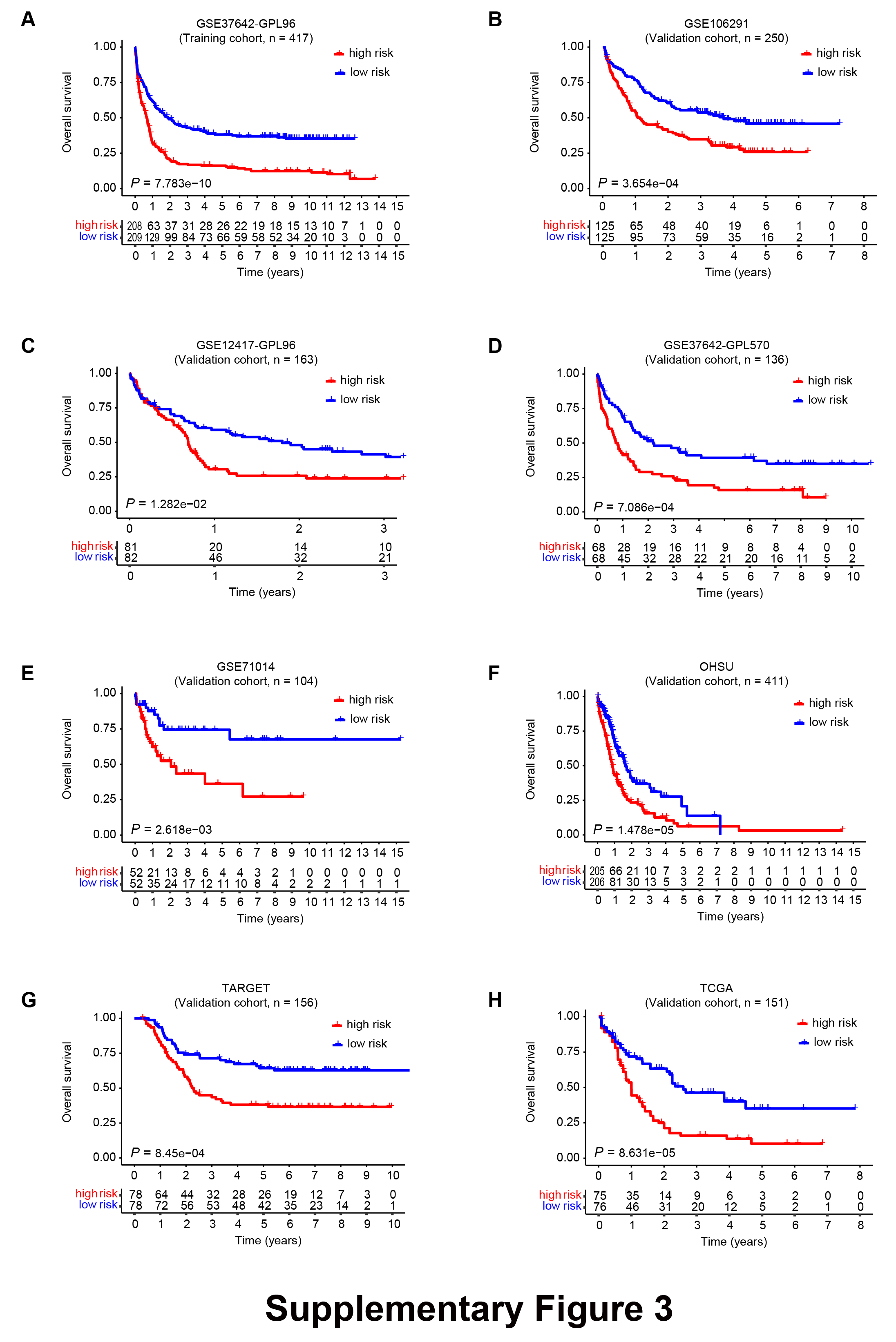

Supplement: Supplementary Figure 3 — Kaplan–Meier survival analyses. (A–H) Kaplan–Meier curves for overall survival stratified by different risk levels in GSE37642-GPL96 (A), GSE106291 (B), GSE12417-GPL96 (C), GSE37642-GPL570 (D), GSE71014 (E), OHSU (F), TARGET (G), and TCGA (H). [file Image_3.tif]

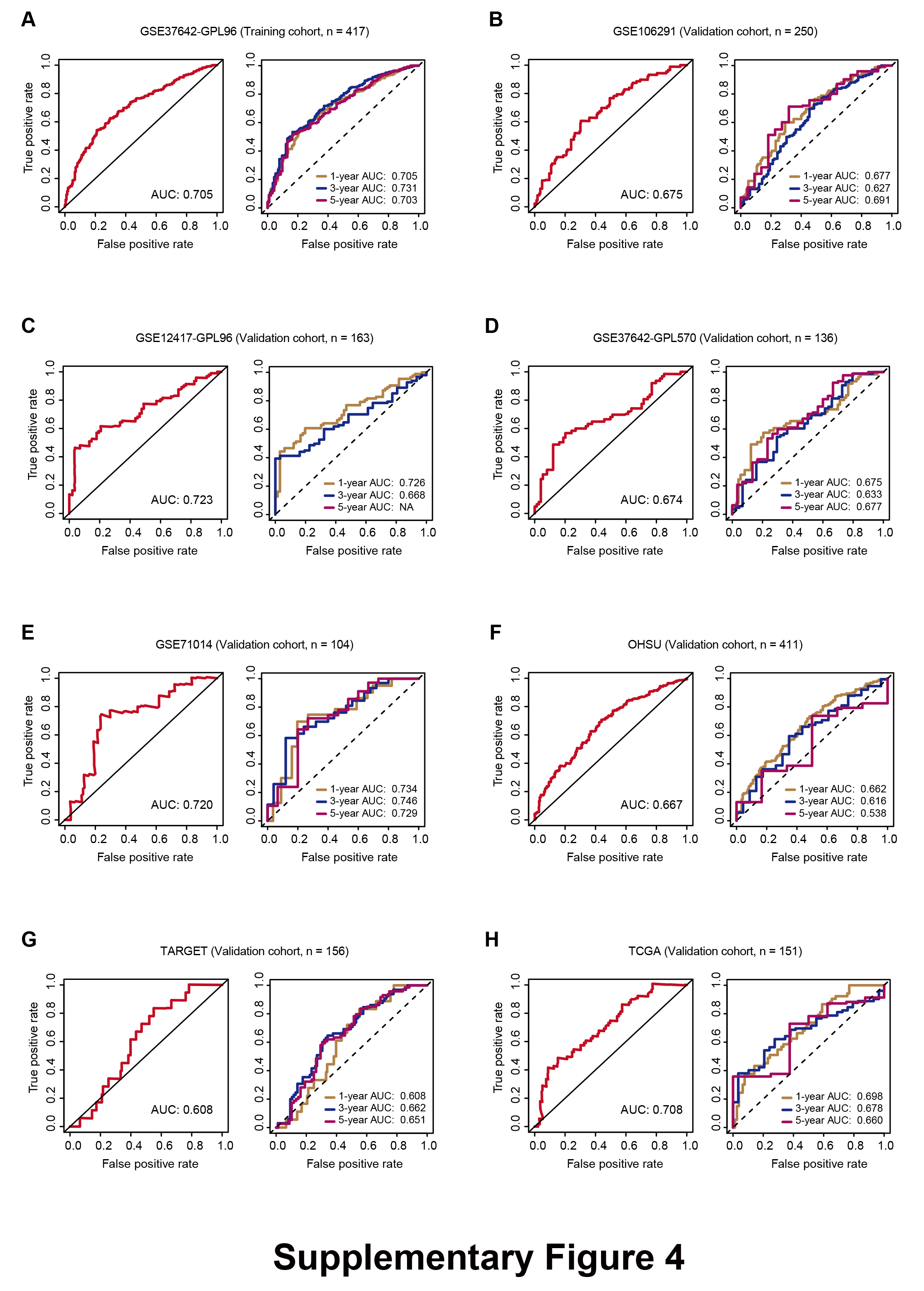

Supplement: Supplementary Figure 4 — Time-independent and time-dependent ROC analyses. (A–H) AUC of time-independent (left) and time-dependent (right) ROC curves of STAT5-associated signature in GSE37642-GPL96 (A), GSE106291 (B), GSE12417-GPL96 (C), GSE37642-GPL570 (D), GSE71014 (E), OHSU (F), TARGET (G), and TCGA (H). [file Image_4.tif]

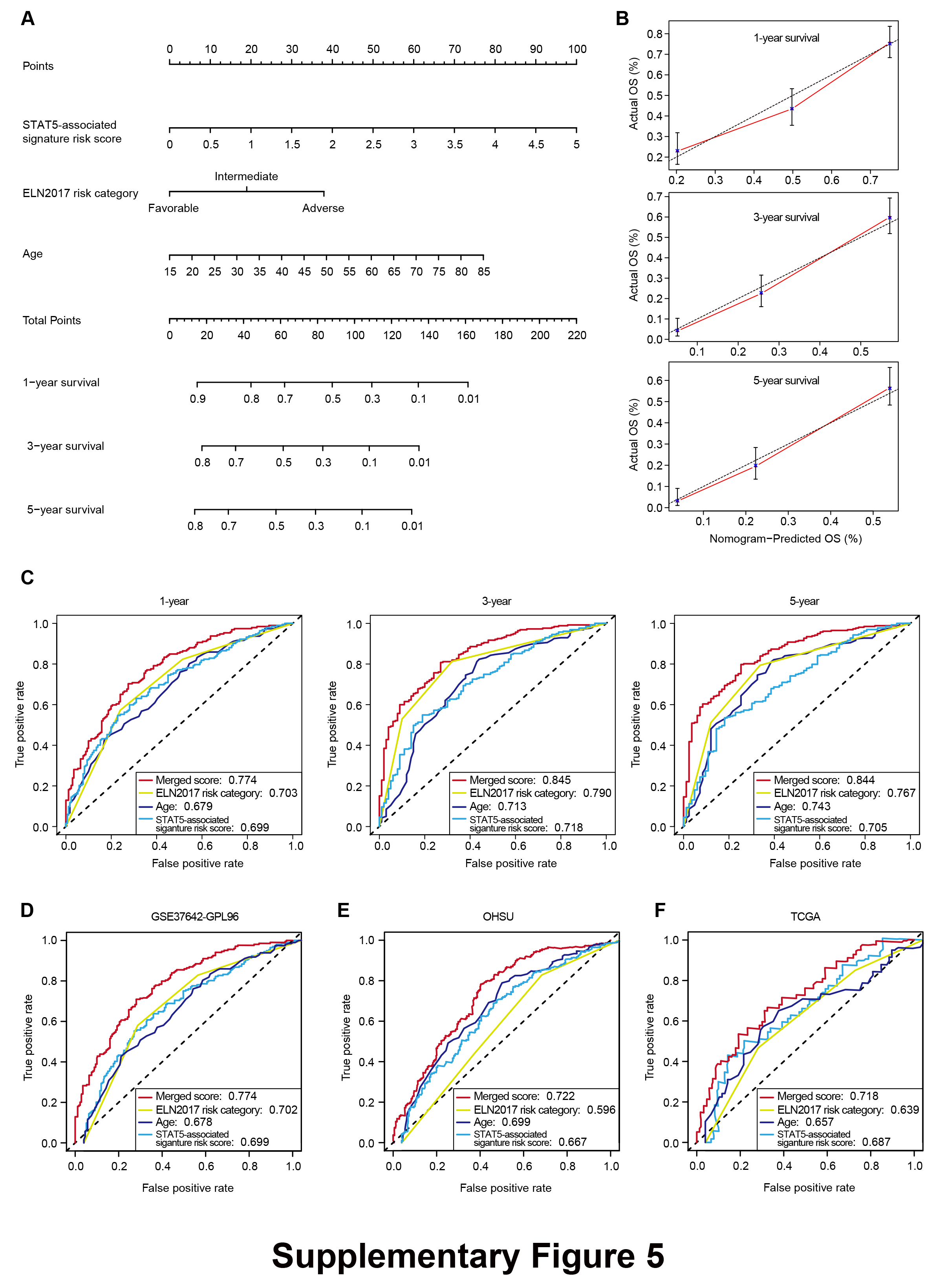

Supplement: Supplementary Figure 5 — Construction of an integrated risk score. (A) Nomogram visualizing the integrated risk model constructed based on the STAT5-associated signature risk score, patient age, and ELN2017 risk category in the training cohort (GSE37642-GPL96). (B) Calibration curves of the nomogram in terms of agreement between predicted and observed 1-year, 3-year, 5-year survival in the training cohort (GSE37642-GPL96). Error bars represent actual overall survival probability with 95% confidence intervals (CI). (C) Comparison of the time-dependent ROC curves of the integrated risk score and its component single risk categories in the training cohort (GSE37642-GPL96). (D–F) Comparison of the time-independent ROC curves of the integrated risk score and its component single risk categories in GSE37642-GPL96 (D), OHSU (E), and TCGA (F). [file Image_5.tif]

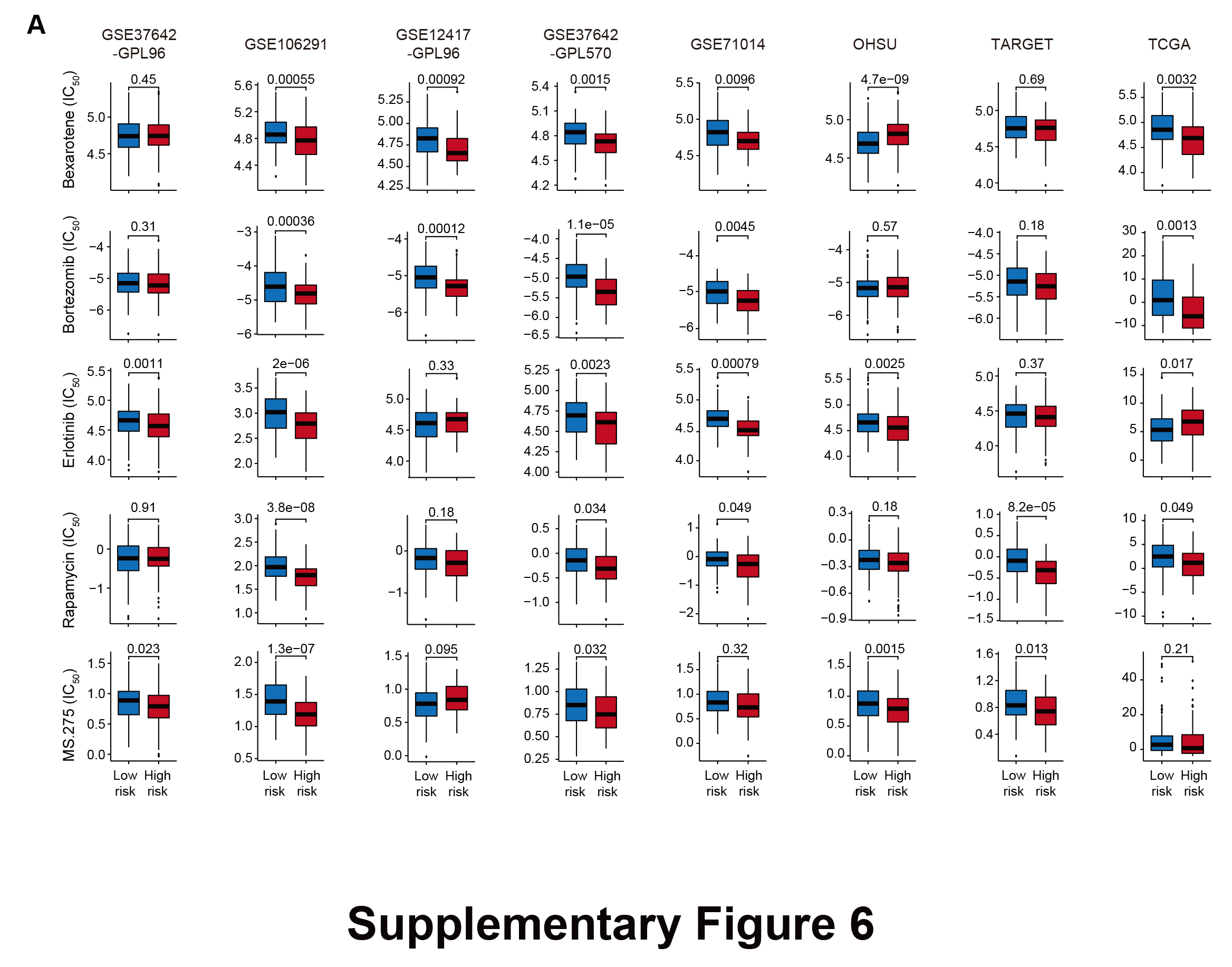

Supplement: Supplementary Figure 6 — In silico screening of chemotherapy drugs for treatment of high-risk AML patients. (A) Estimated IC50 for the five hits from in the indicated cohorts. [file Image_6.tif]

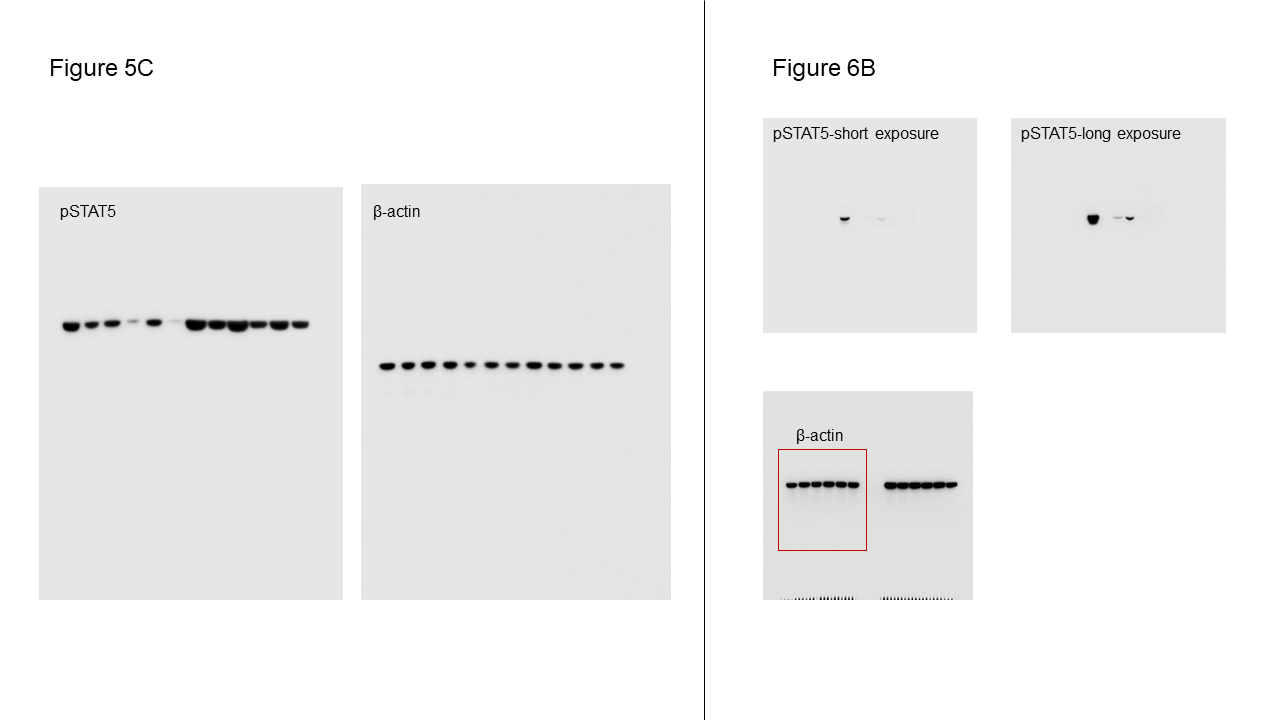

Supplement: Supplementary file 7 [file Image_7.tif]
